# Color Changes in Electronic Endoscopic Images Caused by Image Compression

**DOI:** 10.1155/DTE.4.43

**Published:** 1997

**Authors:** O. Katayama, S. Ishihama, K. Namiki, I. Ohi

**Affiliations:** 1 Clinical Laboratory Saitamaken-Saiseikai Kurihashi Hospital Saitama 349-11 Japan; 2 Endoscope Technical Division Toshiba Medical Co. Tokyo 113 Japan; 3 Central Clinical Laboratory Tokyo Women's Medical College Daini Hospital Tokyo 116 Japan; 4 Kouemon 714-6 Kitakatsushika-gun Kurihashi-cho Saitama 349-11 Japan

## Abstract

In recent years, recording of color still images into magneto–optical video disks has been increasingly used as a method for recording electronic endoscopic images. In this case, image compression is often used to reduce the volume and cost of recording media and also to minimize the time required for image recording and playback. With this in mind, we recorded 8 images into a magneto-optical video disk in 4 image compression modes (no compression, weak compression, moderate compression, and strong compression) using the Joint Photographic Image Coding Experts Group (JPEG) system, which is a widely used and representative method for compressing color still images, in order to determine the relationship between the degree of image compression and the color information in electronic endoscopic images. The acquired images were transferred to an image processor using an offline system. A total of 10 regions of interest (ROls) were selected, and red (R), green (G), and blue (B) images were obtained using different compression modes. From histograms generated for these images, mean densities of R, G, and B in each ROI were measured and analyzed. The results revealed that color changes were greater for B, which had the lowest density, than for R or G as the degree of compression was increased.


*Diagnostic and Therapeutic Endoscopy*, Vol. 4, pp. 43–50
Reprints available directly from the publisher
Photocopying permitted by license only

© 1997 OPA (Overseas Publishers Association)
Amsterdam B.V. Published in The Netherlands
by Harwood Academic Publishers
Printed in Singapore

### Color Changes in Electronic Endoscopic Images Caused by Image Compression

O. KATAYAMA[a,*], S. ISHIHAMA[b], K. NAMIKI[a] and I. OHI[c]

[a]*Clinical Laboratory, Saitamaken-Saiseikai Kurihashi Hospital, Saitama 349-11, Japan;* [b]*Endoscope Technical Division, Toshiba Medical Co., Tokyo 113, Japan;* [c]*Central Clinical Laboratory, Tokyo Women's Medical College Daini Hospital, Tokyo 116, Japan*

*(Received 21 January 1997; In final form 4 March 1997)*

**In recent years, recording of color still images into magneto-optical video disks has been increasingly used as a method for recording electronic endoscopic images. In this case, image compression is often used to reduce the volume and cost of recording media and also to minimize the time required for image recording and playback. With this in mind, we recorded 8 images into a magneto-optical video disk in 4 image compression modes (no compression, weak compression, moderate compression, and strong compression) using the Joint Photographic Image Coding Experts Group (JPEG) system, which is a widely used and representative method for compressing color still images, in order to determine the relationship between the degree of image compression and the color information in electronic endoscopic images. The acquired images were transferred to an image processor using an offline system. A total of 10 regions of interest (ROIs) were selected, and red (R), green (G), and blue (B) images were obtained using different compression modes. From histograms generated for these images, mean densities of R, G, and B in each ROI were measured and analyzed. The results revealed that color changes were greater for B, which had the lowest density, than for R or G as the degree of compression was increased.**

*Keywords:* Electronic endoscope, Image analysis, Image processing, Image compression, Magneto-optical video disk

### INTRODUCTION

As electronic endoscopes (Classen and Phillip, 1984) have increased in popularity, an increasing number of medical institutions have begun to employ various new recording media, such as color video printers, video cassette recorders (VCRs), and magneto-optical video disks, to replace conventional photographic film (Katayama *et al.*, 1995). In practice, image compression is frequently used to increase the efficiency of electronic storage in magneto-optical video disks and other digital media. We previously reported that patterns extracted through image analysis of the

*Corresponding author. Kouemon 714-6, Kurihashi-cho, Kitakatsushika-gun, Saitama 349-11, Japan. Tel.: 0480-52-3611. Fax: 0480-52-1348.

same images differed slightly according to the compression ratio when these images were recorded at different compression ratios using the Joint Photographic Image Coding Experts Group (JPEG) system, which is a representative method for color still image compression (Katayama *et al.*, 1994). In the present study, mean densities of red (R), green (G), and blue (B) were compared between images obtained using different compression modes to assess the effects of image compression on color information in electronic endoscopic images.

### MATERIALS AND METHODS

Eight images were obtained from 6 patients who were randomly selected from among patients undergoing routine endoscopic examination using a scope for the upper gastrointestinal tract with a CCD incorporating approximately 270,000 pixels (TGI-3000D, Toshiba) combined with an electronic endoscope system (TRE-3000, Toshiba) (Yoshie, 1994). These images were recorded in 4 different JPEG image compression modes (no compression, weak compression, moderate compression, and strong compression) using a magneto-optical video disk recorder (MV-200, TEAC). The memory requirements for these 8 images in each compression mode are shown in Table I.

Each of the 8 images recorded in all 4 image compression modes were transferred to an image processor (TOSPIX-U, Toshiba) using an offline system and converted to monochrome images (Fig. 1). After this, 1 or 2 regions of interest (ROIs) were selected in each image, for a total of 10 ROIs (Fig. 2). Then, R, G, and B images were recorded in the 4 image compression modes and histograms of these images were obtained for each ROI (Fig. 3). Using these histograms, the mean densities of R, G, and B in each ROI were calculated (Fig. 4).

### RESULTS

Mean densities of R, G, and B in the 10 ROIs are shown in Table II in the following order: no compression, weak compression, moderate compression, and strong compression. The densities (mean ± SD) of R, G, and B in the 10 ROIs recorded in weak, moderate, and strong compression modes are shown in Fig. 5. R and B showed the highest and lowest densities, respectively. Next, the weak compression/no compression, moderate compression/no compression, and strong compression/no compression ratios of the densities in the 10 ROIs were calculated (mean ± SD) to compensate for the differences in area between the 10 ROIs (Table III). The density

TABLE I Memory requirements for 8 images recorded using magneto-optical video disk recorder MV-200 according to image compression mode

| Endoscopic image | Image compression mode | | | |
|---|---|---|---|---|
| | None | Weak | Moderate | Strong |
| a. DU (Healing) | 1210880 (1/1) | 73594 (1/16) | 52291 (1/23) | 31931 (1/37) |
| b. Atrophic border | 1210880 (1/1) | 81838 (1/15) | 56258 (1/22) | 33874 (1/36) |
| c. DU (Scar) | 1210880 (1/1) | 67978 (1/18) | 45057 (1/27) | 27694 (1/44) |
| d. Gastric polyp | 1210880 (1/1) | 87217 (1/13) | 61347 (1/19) | 38649 (1/31) |
| e. Superf gastritis | 1210880 (1/1) | 74418 (1/16) | 50826 (1/23) | 30583 (1/39) |
| f. EG junction | 1210880 (1/1) | 77980 (1/15) | 54294 (1/22) | 33224 (1/36) |
| g. Duodenal bulb | 1210880 (1/1) | 89835 (1/13) | 62877 (1/19) | 38154 (1/31) |
| h. EG junction | 1210880 (1/1) | 71577 (1/16) | 49997 (1/24) | 30583 (1/39) |

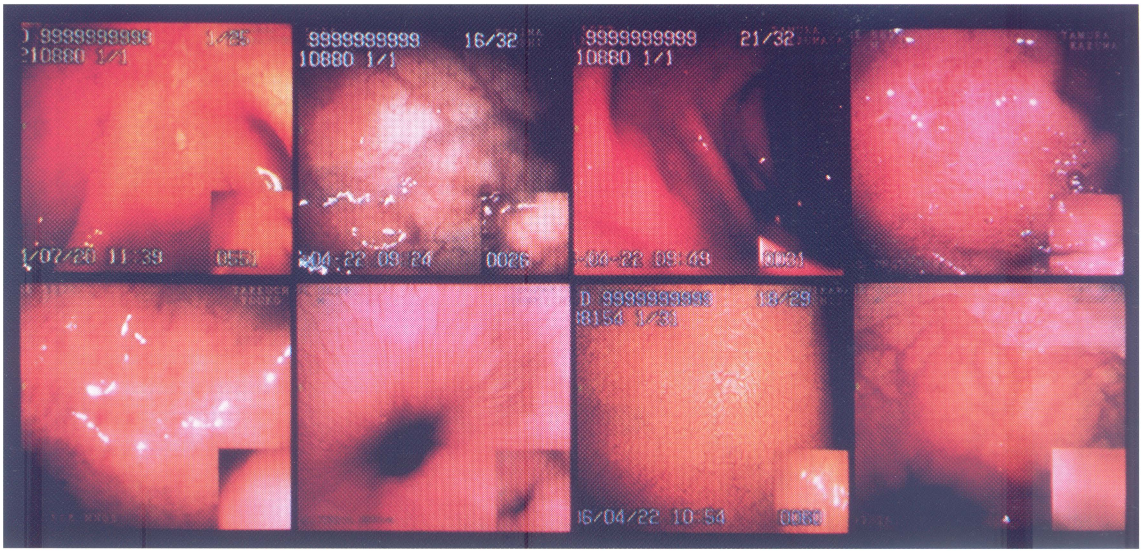

FIGURE 1 Eight endoscopic images transferred to a TOSPIX-U.

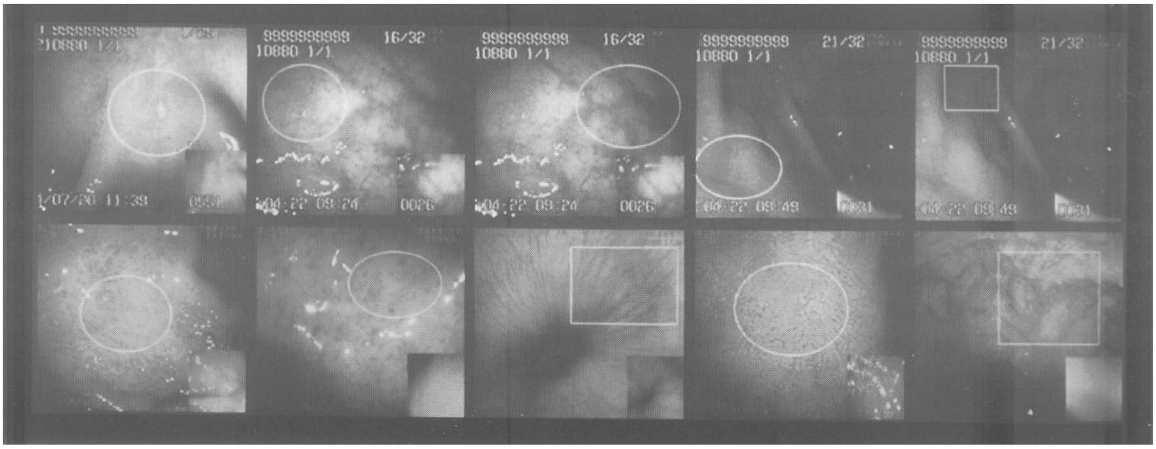

FIGURE 2 Monochrome images in which a total of 10 ROIs were selected for 8 endoscopic images.

ratios (mean ± SD) of R, G, and B in R, G, and B images, respectively, of the 10 ROIs recorded in weak, moderate, and strong compression modes relative to those recorded without compression are shown in Fig. 6. Differences in density between images recorded without compression and images recorded in different compression modes were smallest for R, indicating that the effect of image compression on R was minimal. No clear trends were observed between weak, moderate, and strong compression modes for R or G. On the other hand, compared with images recorded without compression, the difference in density increased as the compression ratio increased for B, indicating that image compression had a pronounced effect for B.

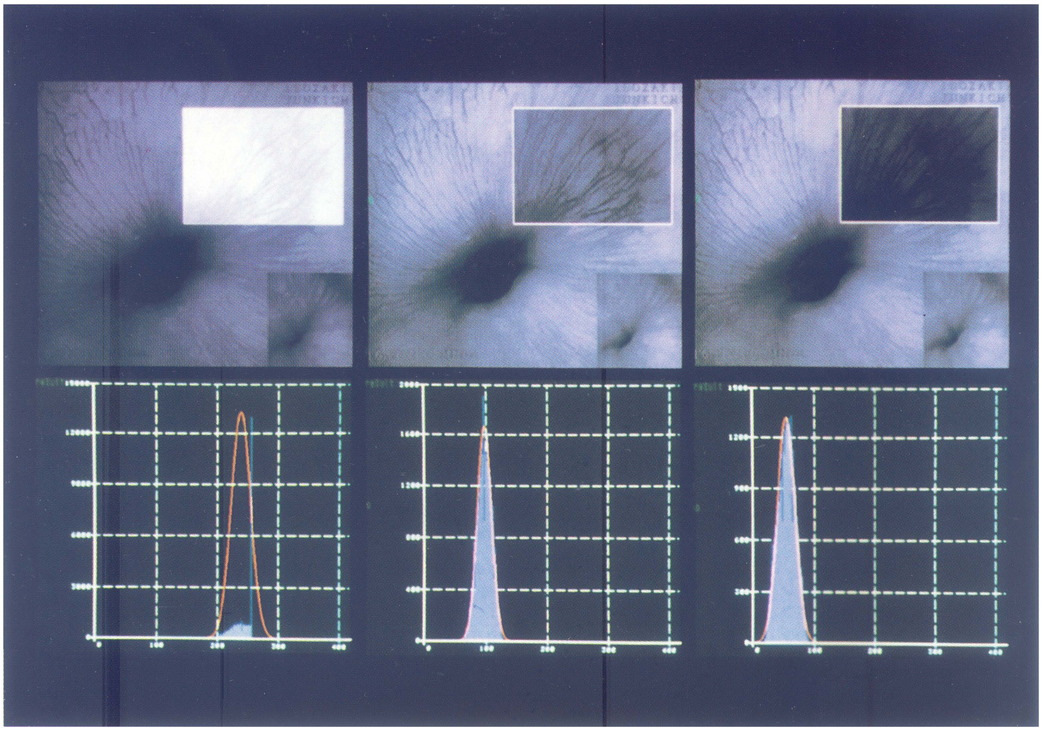

FIGURE 3 Uncompressed images of "f" images (esophagogastric junction) shown in Figs. 1 and 2. R, G, and B images (upper row) and their corresponding histograms (lower row) are shown from left to right.

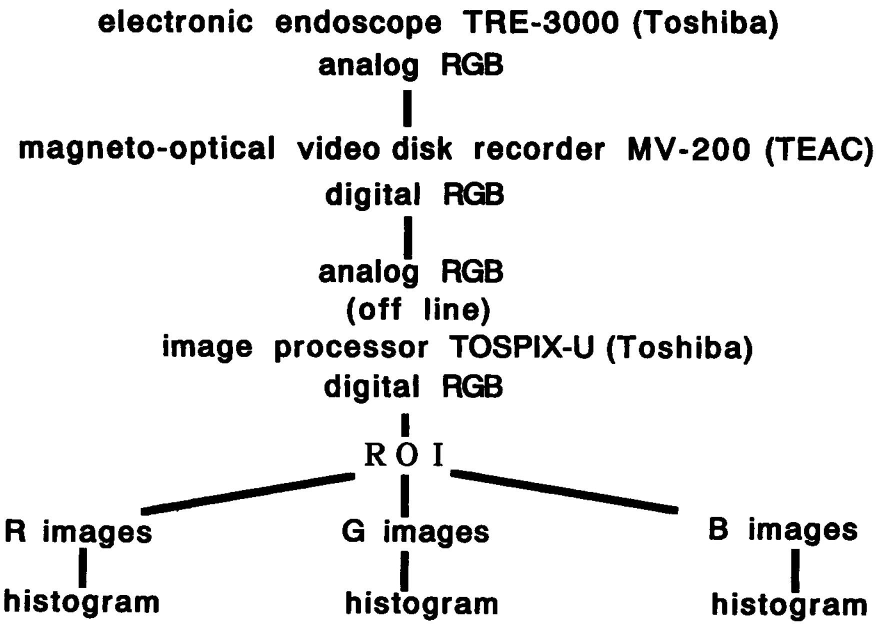

FIGURE 4 Image processing procedure employed in the present study.

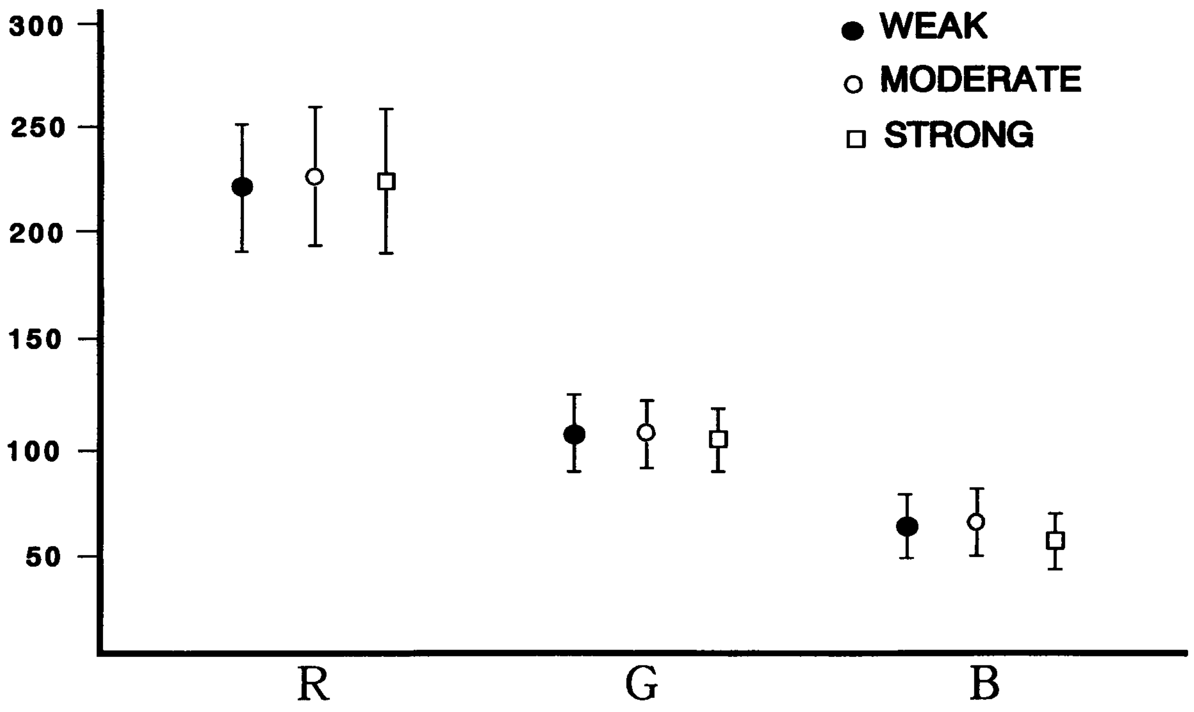

FIGURE 5 Densities (mean ± SD) of R, G, and B in 10 ROIs according to compression mode.

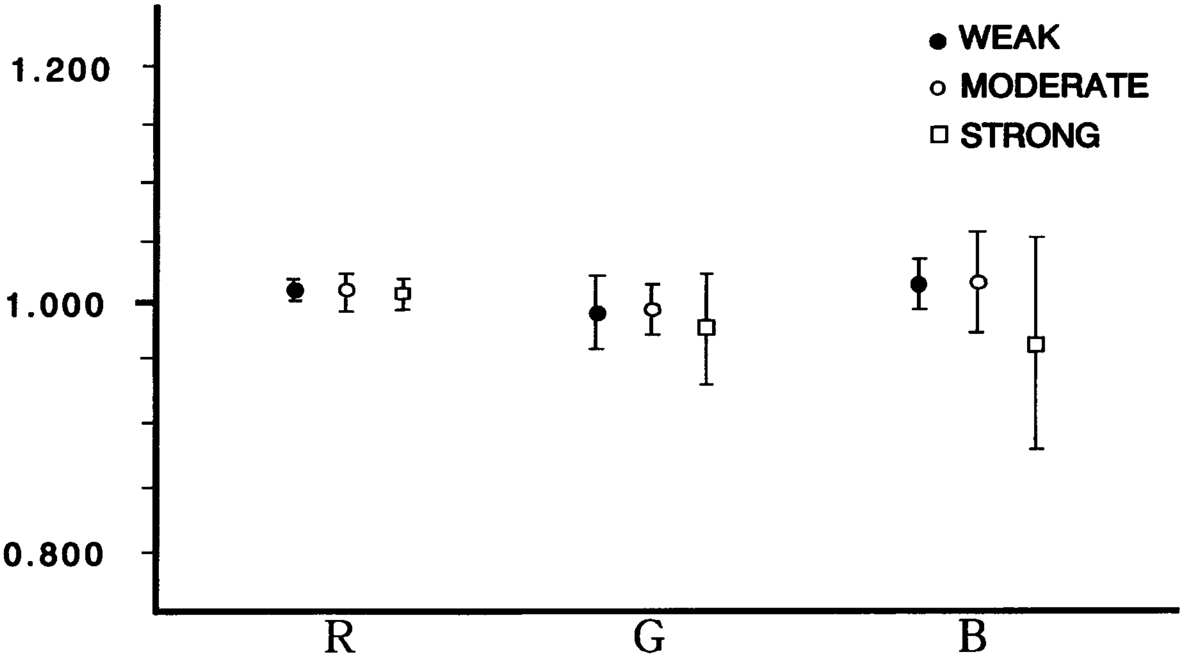

FIGURE 6 Ratios (mean ± SD) of mean densities of R, G, and B in 10 ROIs recorded in weak, moderate, and strong compression modes relative to those recorded without compression.

TABLE II-1 Mean densities of R in R images (according to compression modes)

| | None | Weak | Moderate | Strong |
|---|---|---|---|---|
| a | 250 | 249 | 247 | 249 |
| bl | 216 | 217 | 218 | 215 |
| b2 | 164 | 168 | 170 | 167 |
| cl | 240 | 241 | 241 | 241 |
| c2 | 151 | 152 | 153 | 153 |
| d | 254 | 254 | 254 | 255 |
| e | 242 | 242 | 243 | 242 |
| f | 238 | 240 | 240 | 240 |
| g | 249 | 248 | 248 | 247 |
| h | 239 | 240 | 240 | 240 |
| mean | | 224 | 225 | 225 |
| SD | | 31.8 | 35.3 | 35.9 |

TABLE II-2 Mean densities of G in G images (according to compression modes)

| | None | Weak | Moderate | Strong |
|---|---|---|---|---|
| a | 113 | 114 | 108 | 109 |
| bl | 123 | 120 | 123 | 109 |
| b2 | 105 | 104 | 107 | 95 |
| cl | 91 | 85 | 90 | 91 |
| c2 | 63 | 59 | 62 | 64 |
| d | 116 | 117 | 117 | 116 |
| e | 116 | 117 | 117 | 114 |
| f | 96 | 98 | 99 | 100 |
| g | 128 | 124 | 123 | 122 |
| h | 124 | 124 | 124 | 124 |
| mean | | 106 | 107 | 104 |
| SD | | 20.6 | 19.3 | 18.1 |

TABLE II-3 Mean densities of B in B images (according to compression modes)

| | None | Weak | Moderate | Strong |
|---|---|---|---|---|
| a | 69 | 70 | 63 | 66 |
| bl | 79 | 80 | 81 | 63 |
| b2 | 61 | 65 | 66 | 49 |
| cl | 60 | 60 | 60 | 60 |
| c2 | 26 | 27 | 27 | 27 |
| d | 78 | 80 | 80 | 81 |
| e | 65 | 66 | 67 | 66 |
| f | 54 | 55 | 55 | 55 |
| g | 62 | 60 | 59 | 59 |
| h | 70 | 71 | 71 | 72 |
| mean | | 63 | 64 | 60 |
| SD | | 15.2 | 16.7 | 14.4 |

TABLE III Ratios of densities of R, G, and B in R, G, and B images, respectively, recorded in weak (W), moderate (M), and strong (S) compression modes relative to those recorded without compression (N)

| | R images | | | G images | | | B images | | |
|---|---|---|---|---|---|---|---|---|---|
| | W/non | M/non | S/non | W/non | M/non | S/non | W/non | M/non | S/non |
| a | 0.998 | 0.990 | 0.997 | 1.011 | 0.961 | 0.968 | 1.006 | 0.908 | 0.955 |
| b1 | 1.006 | 1.007 | 0.997 | 0.970 | 0.995 | 0.883 | 1.021 | 1.024 | 0.796 |
| b2 | 1.027 | 1.035 | 1.019 | 0.990 | 1.022 | 0.905 | 1.057 | 1.073 | 0.809 |
| c1 | 1.002 | 1.002 | 1.002 | 0.937 | 0.985 | 1.003 | 1.001 | 1.001 | 1.001 |
| c2 | 1.006 | 1.010 | 1.012 | 0.945 | 0.992 | 1.010 | 1.022 | 1.032 | 1.031 |
| d | 1.000 | 1.000 | 1.000 | 1.009 | 1.005 | 1.003 | 1.018 | 1.020 | 1.028 |
| e | 1.001 | 1.003 | 1.001 | 1.008 | 1.005 | 0.979 | 1.012 | 1.029 | 1.019 |
| f | 1.005 | 1.006 | 1.005 | 1.020 | 1.026 | 1.034 | 1.017 | 1.022 | 1.018 |
| g | 0.998 | 0.995 | 0.995 | 0.974 | 0.962 | 0.955 | 0.974 | 0.959 | 0.957 |
| h | 1.005 | 1.004 | 1.004 | 1.001 | 1.001 | 1.002 | 1.015 | 1.015 | 1.024 |
| mean | 1.005 | 1.005 | 1.003 | 0.987 | 0.995 | 0.974 | 1.014 | 1.008 | 0.964 |
| SD | 0.002 | 0.011 | 0.007 | 0.027 | 0.021 | 0.046 | 0.020 | 0.043 | 0.085 |

### DISCUSSION

Tremendous advances have been made in image compression technology. Although image compression was initially limited to black-and-white still images, methods for compressing dynamic color images, such as the Moving Pictures Experts Group (MPEG) system, have emerged in recent years. Image compression systems for the recording of color still images, such as the JPEG system used in the present study, have been utilized in various fields. Although electronic filing of medical images has mainly been used in the field of radiography, such as CT, MRI, and NM, a relatively large number of institutions are already using or plan to use electronic filing for endoscopic images. Because image compression is frequently employed in such electronic filing to reduce the volume and cost of recording media and to minimize the time required for image recording and playback, the effects of compression on image quality must be thoroughly investigated.

Color information is critical for electronic endoscopic images, unlike radiographs, and it is difficult to visually distinguish between images compressed at 1/30 to 1/40 using the JPEG system, which is utilized in the MV-200 and was employed in the present study, and images recorded without compression. However, there should be some differences between images acquired using the MV-200 at different mean compression ratios, such as 1/10 (weak compression), 1/20 (moderate compression), and 1/30 (strong compression). In fact, the results of the present study indicate that the difference in the density of B, which was lower than that of R or G (Miyahara *et al.*, 1989), relative to that in images without compression, increased as the degree of compression was increased. Thus, image compression using the JPEG system has only minimal effects on R and G, which have high mean densities in electronic endoscopic images, but pronounced effects on B, which has a relatively low mean density. However, since the absolute density of B is small, image compression at 1/30 has only minimal effects on colors in electronic endoscopic images, and the resulting color images are thought to be visually indistinguishable from electronic endoscopic images recorded without compression. In addition, although Yamada *et al.* stated that image processing and image analysis of compressed images suffers from no practical limitations because optimal photographic conditions, such as lighting, are not always achieved in endoscopic images

(Yamada *et al.*, 1990), we should emphasize that the images selected for image analysis in the present study had a high signal level because they were obtained from a short distance from a nearly front view (Katayama *et al.*, 1989) using an electronic endoscope equipped with a CCD incorporating 270,000 pixels (Katayama *et al.*, 1993), and that these images were far superior to those obtained using a conventional endoscope providing only several tens of thousands of effective pixels.

### *References*

Classen, M., Phillip, J. Electronic endoscopy of the gastrointestinal tract, initial experience with a new type of endoscope that has no fiberoptic bundle for imaging. *Endoscopy* 1984; **16**: 16–19.

Katayama, O., Ichioka, S., Hasegawa, M., *et al.* Review of electronic endoscopic finding for image processing. *Prog. Dig. Endosc.* 1989; **35**: 98–100, 408.

Katayama, O., Oguri, K., Ohkubo, Y., *et al.* Usefulness of digital band-pass filter processing by an electronic endoscope that employs a CCD with about 270,000 pixels. *Prog. Dig. Endosc.* 1993; **42**: 51–53, 320.

Katayama, O., Oguri, K., Ohkubo, Y., *et al.* Fundamental study of a pattern extraction method for electronic endoscopic images, evaluation of image compression by edge detection. *Image Technology and Information Display* 1994; **26**: 1069–1074.

Katayama, O., Namiki, K., Kato, A., *et al.* Clinical evaluation of still images generated using the editing, display, and recording function "Muti-Image" of the electronic endoscope. *Medical Review* 1995; **58**: 2–6.

Miyahara, T., Nagao, S., Doi, T., *et al.* Capability of each spectrum of visible rays representing upper gastrointestinal disease. *Gastroenterol Endosc.* 1989; **31**: 2595–2604, 2613.

Yamada, Y., Suzuki, S., Shiina, Y., *et al.* Development of digital filing system for electronic endoscopic images with image compression by discrete cosine transform. *Gastroenterol Endosc.* 1990; **32**: 2189–2197.

Yoshie, T. TV endoscope system, TRE-3000. *Medical Review* 1994; **52**: 25–29.

